# Real-world effectiveness of pneumococcal vaccination in older adults: Cohort study using the UK Clinical Practice Research Datalink

**DOI:** 10.1371/journal.pone.0275642

**Published:** 2022-10-13

**Authors:** Adam J. Streeter, Lauren R. Rodgers, Jane Masoli, Nan X. Lin, Alessandro Blé, Willie Hamilton, William E. Henley

**Affiliations:** 1 Institute for Epidemiology and Social Medicine, University of Münster, Münster, North Rhine-Westphalia, Germany; 2 Medical Statistics, Faculty of Health, University of Plymouth, Plymouth, United Kingdom; 3 Institute for Health Research, University of Exeter Medical School, University of Exeter, Exeter, United Kingdom; 4 Department of Mathematics, Physics and Electrical Engineering, Northumbria University, Newcastle upon Tyne, United Kingdom; University of Phayao, THAILAND

## Abstract

**Background:**

The 23-valent pneumococcal polysaccharide vaccine (PPV23) is recommended for UK older adults, but how age moderates effectiveness is unclear.

**Methods:**

Three annual cohorts of primary-care patients aged≥65y from the Clinical Practice Research Datalink selected from 2003–5 created a natural experiment (n = 324,804), reflecting the staged introduction of the vaccine. The outcome was symptoms consistent with community-acquired pneumococcal pneumonia (CAP) requiring antibiotics or hospitalisation. We used the prior event rate ratio (PERR) approach to address bias from unmeasured confounders.

**Results:**

Vaccinated patients had higher rates of CAP in the year before vaccination than their controls, indicating the potential for confounding bias. After adjustment for confounding using the prior event rate ratio (PERR) method, PPV23 was estimated to be effective against CAP for two years after vaccination in all age sub-groups with hazard ratios (95% confidence intervals) of 0.86 (0.80 to 0.93), 0.74 (0.65 to 0.85) and 0.65 (0.57 to 0.74) in patients aged 65–74, 75–79 and 80+ respectively in the 2005 cohort. Age moderated the effect of vaccination with predicted risk reductions of 8% at 65y and 29% at 80y.

**Conclusions:**

PPV23 is moderately effective at reducing CAP among UK patients aged≥65y, in the two years after vaccination. Vaccine effectiveness is maintained, and may increase, in the oldest age groups in step with increasing susceptibility to CAP.

## Introduction

Pneumonia is a major cause of morbidity, hospitalization and associated mortality in older adults [1]. Since 2003, public health policy in the UK has recommended vaccination against streptococcus pneumoniae (pneumococcus) for adults aged ≥65y using the 23-valent polysaccharide pneumococcal vaccine (PPV23). The vaccination programme began in August 2003 with the PPV23 vaccine offered to adults aged ≥ 80y. This was extended to adults aged ≥ 75y in April 2004 and then finally to all adults aged ≥65y in April 2005. PPV23 is recommended as a standard intervention for the elderly in many other countries across Europe and elsewhere. However, more data on PPV23 in older adults is needed while vaccination rates remain below target levels and changes in effectiveness with age among older age groups remain poorly understood [2]. Age-related decline in immune function may reduce the immunogenic response to vaccination, but it is not known by how much this may reduce effectiveness. Protection against pneumococcus may be more crucial amongst the oldest old, given rising susceptibility with age to pneumococcal infection. Four systematic reviews have been published since 2016 with divergent conclusions [3–8], and recently published evidence from observational data [9, 10], relying on older analytic methods [11] might not fully account for bias from unmeasured sources [12].

The Community-Acquired Pneumonia Immunization Trial in Adults (CAPITA), a large-scale, population-based randomised conducted in the Netherlands, reported an efficacy of 46% against first episodes of vaccine-matched strains of community-acquired pneumonia and 75% against invasive pneumococcal disease among 84 496 adults aged ≥65y [13] with no apparent waning in efficacy over the four year follow-up. However, the study lacked the power to draw conclusions on how efficacy might vary with the age of the vaccine recipient. Furthermore, the intervention was protein-conjugated polysaccharides from 13 serotypes (PCV13), a vaccine originally developed for young children but licensed since for use in adults primarily on the basis of immunogenicity studies. The effectiveness of PPV23, the vaccine offered to adults in many countries including the UK, has been reported for adults aged ≥65y in two studies using the test-negative case-control design. Vaccine effectiveness against all-cause pneumonia and vaccine-matched strains, respectively, was reported to be 23% and 34% in the study by Suzuki et al. [14]. In the study by Lawrence et al. [15], the adjusted vaccine effectiveness against vaccine-matched strains was reported to be 20%. However, without randomisation, both trials relied on a test-negative case-control design to mitigate against confounding. Suzuki et al., found effectiveness appeared to wane with time since vaccination, but the precision was too low to be definitive, while Lawrence et al. found this was maintained. The reported decrease in effectiveness with age in both studies was imprecise and not supported by statistical evidence.

To determine how effectiveness might change with age among older adults, we conducted a retrospective cohort study using electronic health records (EHRs) to assess real-world effectiveness in UK adults aged ≥65y. The steep rise in vaccination rates resulting from the introduction of the vaccination programme for older adults from 2003 to 2005 provided the opportunity for a natural experiment and the incremental introduction by age group facilitated estimation of vaccine effectiveness within the key age sub-groups. The data were extracted from the Clinical Practice Research Datalink (CPRD) with linkage to Hospital Episode Statistics and Office of National Statistics data. Large EHR databases can afford larger sample sizes for the study of real-world effectiveness in small sub-groups than would typically be available in randomized trials, as well as facilitating the study of populations which, for ethical reasons, might otherwise be difficult to recruit into a trial.

## Methods

### Data and study design

We used routinely-collected electronic patient records from the UK Clinical Practice Research Datalink (CPRD) [16].

**Fig 1:**
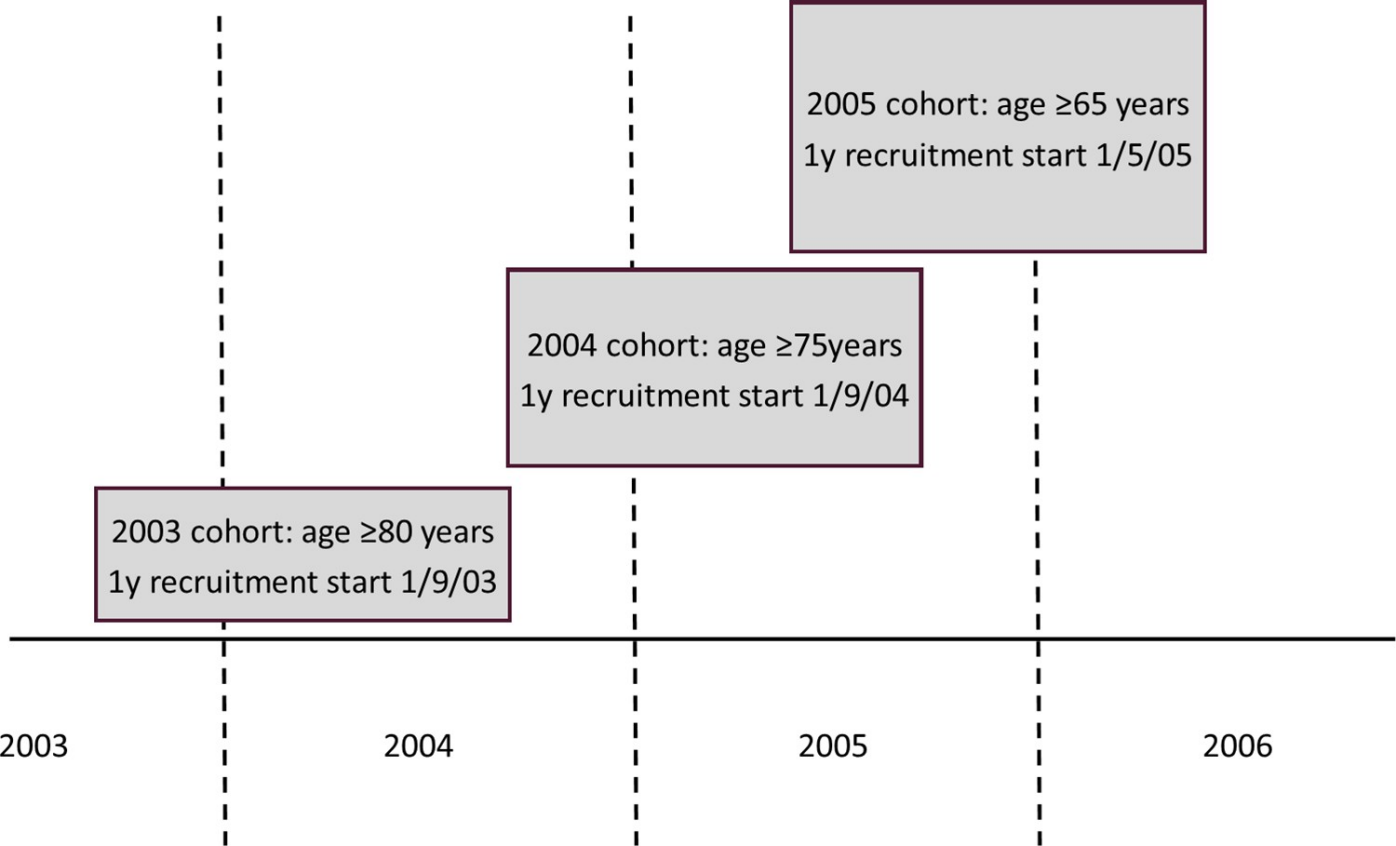
Diagram outlining recruitment of the three cohorts coinciding with stepped implementation of the policy to offer the vaccine to older adults, beginning with ≥80y in 2003, then additionally the ≥75y age group and finally all adults aged ≥65y.

Three cohorts were studied, each relating to a single year of the phased introduction of PPV23 by age-group. The start and end dates for recruitment were chosen to capture peak uptake of vaccination near the beginning of the period: adults aged ≥80y, vaccinated for the first time from 1^st^ September 2003 to 31^st^ August 2004; adults aged ≥ 75y from 1^st^ September 2004 to 31^st^ August 2005; and adults aged ≥65y from 1^st^ May 2005 to 30^th^ April 2006. The data comprised three age groups ≥80y (all cohorts), 75-79y (2004 and 2005), and 65-74y (2005 only) (Fig 1). Patients had to be alive and registered at their general practice at the (index) date of vaccination. All adults that remained unvaccinated for the duration of the study period were designated as controls and matched to vaccinees, to the nearest age, and where possible the same gender and practice, solely for the purposes of assigning an index date rather than adjusting for confounding bias. The index date for each control was the vaccination date of the corresponding vaccine recipient. Each cohort was analysed separately. Any patients without data in the six years preceding recruitment were excluded from the cohort to mitigate against inclusion of patients, who had left their practice without de-registration.

**Fig 2:**
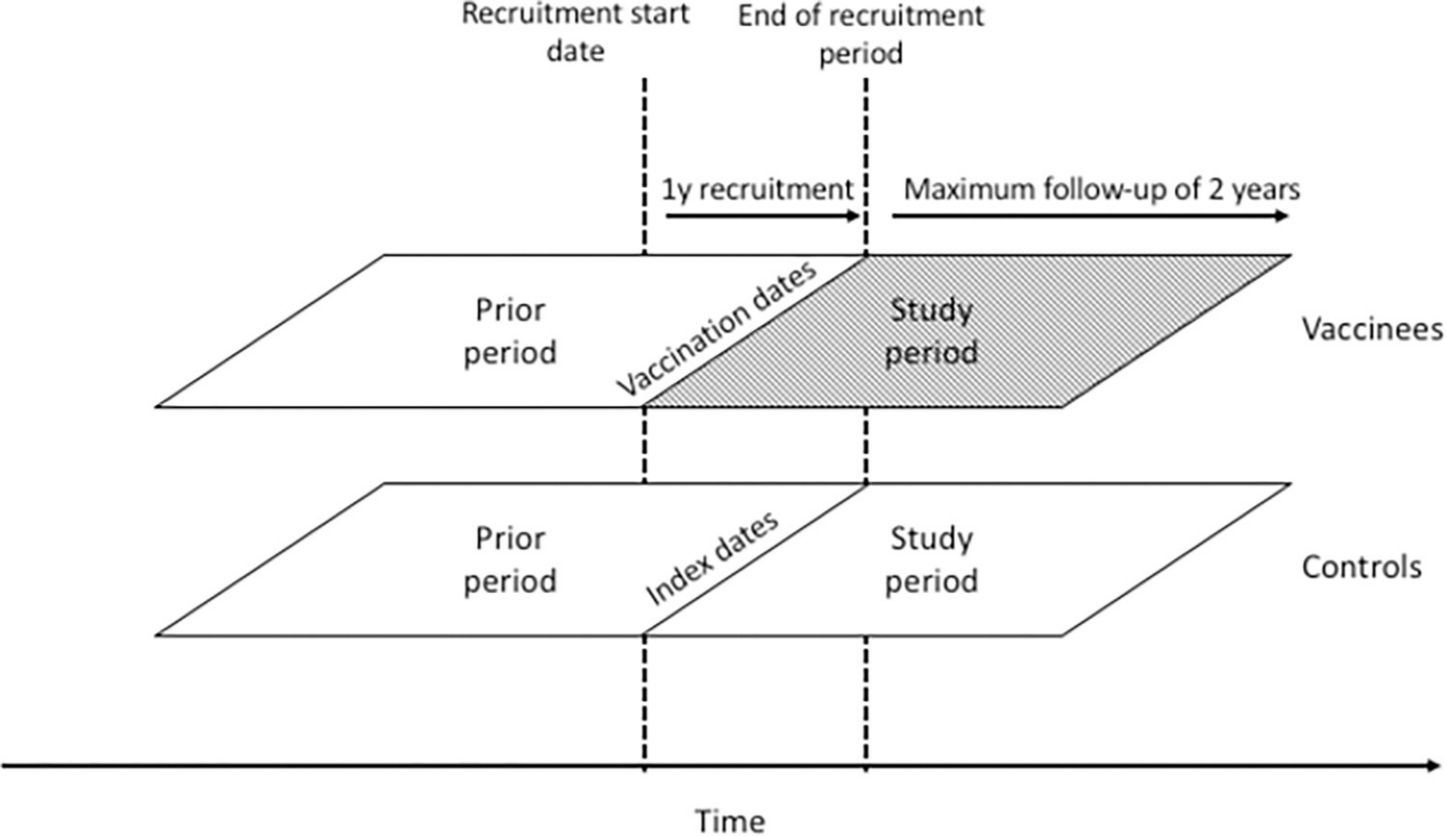
Schematic of the study design. Patients vaccinated during a 1y recruitment window are selected and matched to controls by age, gender and general practice. Index dates of controls are mapped from the vaccination dates of vaccinees. Event times are compared for vaccinated and control patients during a 2y study period and a 2y prior period. The start of the prior period precedes recruitment by exactly 2y. Survival times may end with an event or be censored before the end of either period.

The large uptake in vaccination during the years of policy implementation provided the basis for a natural experiment to adjust for unmeasured components of confounding in a two arm before-and-after study design. Patients were followed for up to two years from the index date, censoring on death or de-registration from their practice. A two year study follow-up period was the time interval over which the effect of the vaccine was found to be stable in previous studies [17] suggesting reasonable stability of unmeasured confounding effects over this period. By also collecting data on the two-year period prior to vaccination, during which all patients were unvaccinated, further adjustment could be made for unmeasured confounding through the framework of the prior event rate ratio (PERR) (Fig 2).

### Outcomes and covariates

In the absence of routine testing for pneumococcal pneumonia in suspected cases, we utilized the clinical records to specify a real-world, composite outcome for community-acquired pneumonia (CAP) comprising hospital admissions for suspected pneumococcal pneumonia and the prescription of antibiotics, amoxillin and doxycycline, species typically used for treating pneumonia, both qualified by the coded symptoms consistent with those of the disease [18, 19]. Hospitalisations were coded according to their ICD-10 classification. Patients were identified as being vaccinated with PPV23 using relevant codes in the CPRD immunisation file, supplemented with codes from the therapy file (S1 Appendix).

### Adjustment for unmeasured confounding

Without randomization, vaccination status in observational studies of this type may be influenced by unmeasured confounders. Recent advances in quasi-experimental methods make it possible, under specific assumptions, to address directly bias that arises when relevant confounders are omitted [20, 21]. One such approach to enhancing the validity of observational studies is the Pairwise method [22], an extension by Yu et al. [23] and Lin and Henley [22] to the Prior Event Rate Ratio (PERR) method. The PERR method, originally proposed by Tannen and Weiner et al. [24, 25] is increasingly being used in vaccine effectiveness studies [26–28], and has previously demonstrated that the association between CAP and proton pump inhibitors can be attributed to confounding factors [29]. The PERR method adjusts the estimate for the study period as illustrated in Fig 2 with that from the prior. The Pairwise method makes the adjustment within each exposure group before estimating the adjusted effect of exposure and has been shown to be less sensitive to the presence of hidden covariates and censoring [30].

The moderating effect of age was evaluated in the 2005 cohort by applying the Pairwise method to the analysis of the interaction between age (as a linear effect) and vaccination status, across the full age range of adults≥65y. In a further investigation into whether immunity was maintained over the length of follow-up, we also separately estimated the HR for vaccination against CAP, using the pairwise method, for the first and second year of follow-up by age group in each cohort (S1 Appendix).

### Sensitivity analyses

The robustness of the adjustment for unmeasured confounding was assessed through analysis of a negative control outcome (NCO) (S1 Appendix). A valid NCO is one that shares the same potential sources of bias with the primary outcome but cannot plausibly be related to the treatment of interest. We chose ICD-coded hospital admissions for fractures (excluding thoracic injury) as a viable NCO. We make the assumption that this outcome may be directly caused by confounders for the effect of PPV on CAP, but not directly by CAP or PPV. The analysis of the PPV23 effect on fractures can be used as an indicator of the degree of any remaining bias after the pairwise adjustment.

The results were compared to those explicitly adjusted for measured confounding using inverse probability treatment weighting (IPTW) of the Cox regression models of the times until CAP by age group in each of the cohorts (S1 Appendix). This provided a check on the consistency of results, and thus the plausibility, of age-related effectiveness. We then applied the PERR method as an adjustment for residual confounding in the IPTW models [31–33].

## Results

There was good concordance between the PPV uptake achieved by the end of 2005 in the study data and national vaccination rates reported by Public Health England (PHE—formerly the Health Protection Agency) for uptake by 31^st^ March 2006: In the extracted data, PPV uptake was 64.8%, 70.6%, 68.4% for age groups ≥65y, 75-79y and 80y and older respectively; while PHE presented 64.4%, 68.9%, 68.1%, respectively for the same age groups.

### Cohort characteristics

**Fig 3:**
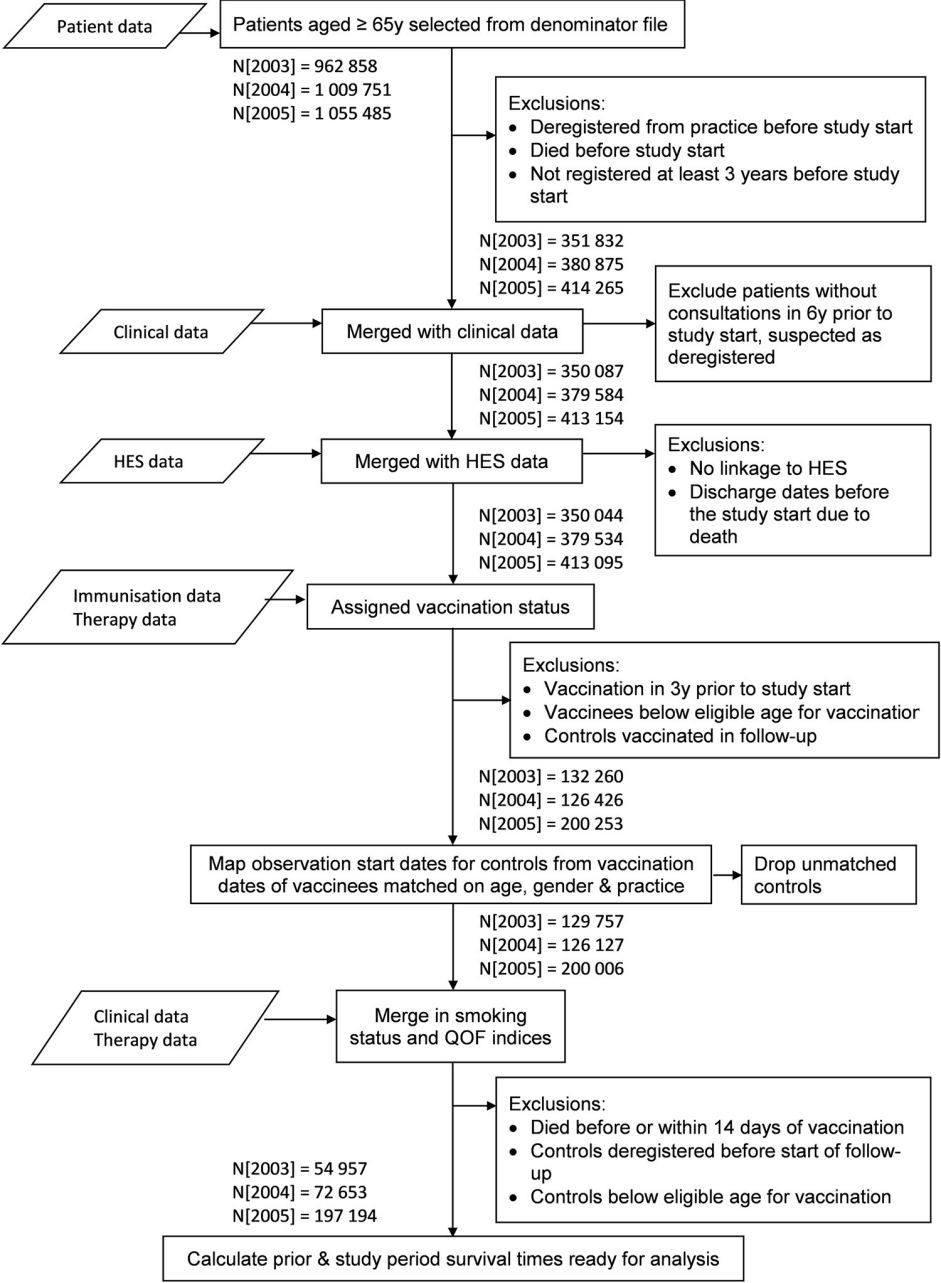
Flowchart for the three cohorts 2003–2005.

Cohort sizes increased with each study year as the vaccination programme was expanded; about half of each cohort comprised vaccinees with 47.1% in 2003, 41.3% in 2004 and 53.2% in 2005. Over 40% of the 2005 cohort were males, decreasing to about a third for the older 2003 cohort (Table 1; flowchart in Fig 3). The controls were at least two years older on average with fewer males, and a lower prevalence of disease, particular cardiovascular (S1 Table).

**Table 1:** Rates of composite CAP outcome, death and, censoring for each cohort from 2003 to 2005.

Cohort		2003		2004		2005	
Treatment group		Vaccinees	Controls	Vaccinees	Controls	Vaccinees	Controls
N		25870	29087	30028	42625	104969	92225
% males		36.7%	28.5%	40.4%	31.8%	44.7%	40.3%
Mean age (SD)		84.5 (4.0)	85.9 (4.8)	79.3 (4.3)	82.3 (5.8)	71.6 (5.4)	75.1 (8.1)
Deaths	% patients censored on death	15.4	31.6	9.1	23.4	4.1	13.3
Transfers out of practice (t.o.)	% patients censored for transferring out of practice	6.4	13.4	4.4	10.3	4.0	8.4
Outcomes in study period	% patients with CAP outcome	10.9	9.7	9.1	8.4	7.3	6.5
	% hospitalised pneumonia cases among outcomes	30.5	48.7	23.5	43.8	13.8	31.6
	% hospitalised pneumonia cases who died during study	68.1	81.3	58.7	75.8	50.0	69.9
Outcomes in prior period	% patients with CAP outcome	8.4	7.3	7.7	6.7	6.7	5.6
	% hospitalised pneumonia cases among outcomes	12.4	24.9	10.1	21.4	6.1	16.4

The overall risk of the composite CAP outcome decreased from 10.2% in the 2003 cohort to 6.9% in the 2005 cohort, reflecting the younger age distribution for the later cohorts (Table 1). Both vaccinated and control patients were more likely to experience a CAP event in the study period than in the prior period. The proportion of hospitalisations for pneumococcal pneumonia among patients experiencing a prior or study end point tended to be greater for the controls, as high as 49% for those in the older 2003 cohort, while 31% for the vaccinees. However, the rates of CAP in the prior period were higher for patients that went on to be vaccinated with PPV23 than for patients who remained unvaccinated, suggesting analysis of the study period could be biased without adjustment. One of the central assumptions of the PERR adjustment approach is that the occurrence of prior events does not influence the likelihood of future treatment. We note that in this study, outcomes in the prior period did not greatly differentiate subsequent vaccination status, at most by 4.8% in 2005 with 57.7% of patients with CAP being vaccinated, compared to 52.9% of those without CAP.

Control patients had higher mortality rates than vaccinated patients with 32% of the controls from the older 2003 cohort being censored on death compared to less than half that figure (15%) among the vaccinees. This imbalance increased with each year of recruitment, a trend that was tempered by the inclusion of younger patients in later cohorts, highlighted by the overall reduction in mortality from 19% to 7% per cohort by 2005. Those hospitalised for pneumonia were at the greatest risk of death, particularly in the older 2003 cohort (68% and 81% following hospital admissions in vaccine recipients and controls, respectively). Consistent with the high mortality rate following pneumonia hospitalisation, the proportion of outcomes resulting in hospitalisation was lower during the prior period than the study period, as patients needed to be alive after the prior period for subsequent selection to the study. In comparison to deaths, there were far fewer censored survival times due to deregistrations from the general practices.

### PPV23 effectiveness by age

The hazard ratios (HRs) from the naïve Cox models (age and sex adjusted) from the study period were equal to 1 for the 2003 cohort, but greater for all age groups in the other cohort indicating an implausible, harmful effect of vaccination (Table 2). However, the HRs from the prior periods were all greater than the corresponding HRs of the study period–evidence of pre-existing confounding bias. Once the study period estimates were adjusted with the difference between the vaccine groups in the prior period through the Pairwise method, all HRs were below unity, indicating a protective effect of PPV23.

**Table 2:** Hazard ratios, adjusted for age and gender, presented for age-groups of the prior and study periods pertaining to each cohort, and their pairwise-adjusted estimates. Age groups, which were incrementally targeted for pneumococcal vaccination from 2003 to 2005, comprised adults aged over 79y; from 75 to 79y; and from 65 to 74y.

Cohort year	Age group	Hazard ratios (95% CI) of Treatment term		
		Prior	Study	Pairwise
2003	80+	1.20 (1.13, 1.27)	1.00 (0.95, 1.06)	0.68 (0.63, 0.74)
2004	75–79	1.23 (1.14, 1.34)	1.12 (1.03, 1.20)	0.82 (0.72, 0.93)
2004	80+	1.34 (1.23, 1.45)	1.07 (0.99, 1.15)	0.61 (0.54, 0.69)
2005	65–74	1.37 (1.30, 1.44)	1.28 (1.22, 1.34)	0.86 (0.80, 0.93)
2005	75–79	1.27 (1.16, 1.39)	1.08 (0.99, 1.17)	0.74 (0.65, 0.85)
2005	80+	1.31 (1.20, 1.42)	1.07 (0.99, 1.15)	0.65 (0.57, 0.74)

**Fig 4:**
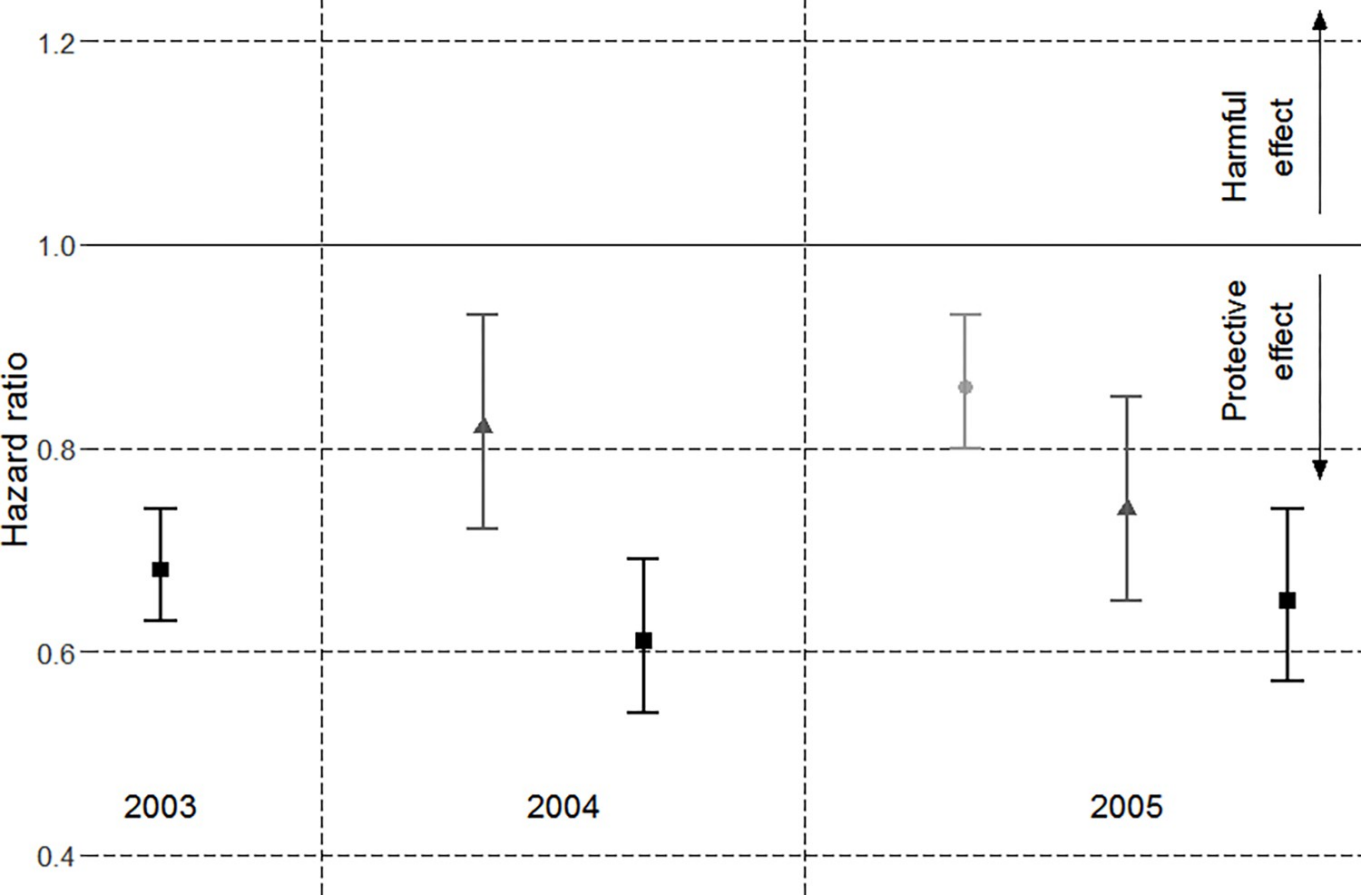
Pairwise-adjusted hazard ratios of vaccination for each annual cohort (2003–005) by sub-groups of age (65 to 74y –light grey circle; 75 to 79y –mid-grey triangles; 80+y–black squares.

There was reasonable concordance between the pairwise-adjusted HRs for the 80+y age group across the cohorts, which varied from 0.61 (95% CI 0.54 to 0.69) in 2004 to 0.68 (95% CI 0.63 to 0.74) in 2003. In both the 2004 and 2005 cohorts, there was a clear trend towards greater effectiveness at older ages. PPV23 was effective against CAP in the 2005 cohort across all age groups with the HR for the 65-74y group at 0.86 (95% CI 0.80 to 0.93), lower for the 75-79y group at 0.74 (95% CI 0.65 to 0.85) and lower still for the 80+y group at 0.65 (95% CI 0.57 to 0.74) (Fig 4).The gradient of age-related effectiveness was greater for the 2004 cohort since the HR for the 75-79y group in the 2004 cohort was greater than the corresponding group in the 2005 group.

In the analysis of the moderating effect of age on vaccination for the 2005 cohort, a significant interaction term identified an increasing protective trend with age (p-value = 0.01). According to the HR of 0.98 (95% CI: 0.98 to 0.99) for the interaction term the estimated reduction in the rate of CAP in the vaccinated patients improved by 8% for every 5 year increase in age. Given the interaction and the HRs for the main effects of age, centered on 65y, (HR = 1.03) and vaccination (HR = 0.92), this equates to a risk reduction of 8% at 65y, 23% at 75y and 29% at 80y.

## Discussion

This study has shown that vaccination with PPV23 is effective in protecting older adults aged 65 and above against pneumococcal community-acquired pneumonia in routine clinical practice. To the best of our knowledge, this is the first population study to establish that vaccine effectiveness is maintained, and may even increase, in the oldest age groups: the reduction in risk due to PPV23 vaccination was estimated to be about 15% in adults aged 65–74 and increased to 35–40% in adults aged 80 or above. This would indicate that while immunogenicity may be thought to weaken with age (immunosenescence), the vaccine can still provide protection against the age-related increase in the risk of CAP. We also found higher rates of comorbidity among vaccinated patients, suggesting vaccine take-up was higher amongst patients in closer contact with the health-care system. The vaccinated, being younger with fewer hospital admissions for CAP and having a lower fracture risk and mortality suggested PPV23 was administered to those more likely to benefit from long-term immunity to pneumococcal disease.

Our study has several strengths: firstly, our data source, the Clinical Practice Research Datalink, with current coverage of about 11 million patients, is representative for the general population of patients in the UK [34]. Using this database and adequate sample selection strengthens generalizability of our findings from 324,804 elderly patients, a population that is typically under-represented in RCTs due to many ethical and logistic barriers in recruitment.

The pairwise method used in this study has previously demonstrated the effectiveness of the influenza vaccine in reducing antibiotic prescribing, adjusting for confounding bias from unmeasured sources [27]. This method, equivalent to the PERR-ALT method, is a recent formulation of the PERR approach, which has also recently been used in respiratory medicine research [26, 29]. The pairwise method overcomes bias by fitting a paired Cox model to the prior and study periods and is less sensitive to bias from censoring and hidden covariates than the PERR. Although the randomized controlled trial (RCT) remains the gold standard for evidence, applying the PERR methods to retrospective cohorts has been shown to reproduce results from RCTs [24, 25, 35, 36].

A key assumption of the PERR and Pairwise approaches is that prior events do not influence the likelihood of future treatment. We found similar vaccination rates in patients with and without a suspected pneumonia event in the prior period suggesting this assumption was likely to be satisfied. A second main assumption required for PERR and Pairwise analyses is the lack of substantive time-dependent confounding. Bias arising from confounding may change due to declining health and increasing frailty in this population. Applying the PERR and Pairwise methods may fail to correct for bias, or even exacerbate the degree of bias, if the confounding bias varies between the prior and the study period. We tried to address this by limiting the follow-up to two years post-vaccination and by replicating results for the 74–79 and 80+ age sub-groups across multiple recruitment cohorts. However, even over a short period of follow-up, there was still a consistent drop in vaccine effectiveness by the second year, across the age groups in each cohort.

While we used the Pairwise method to account for confounding bias, including unmeasured sources, we also conducted a sensitivity analysis to test the validity of our results and robustness of assumptions. Adjusting for measured confounders alone through the weighted analysis suggested the presence of residual confounding, but subsequent adjustment with the PERR method indicated a clearer, protective effect of vaccination. The results were slightly more conservative than those from the Pairwise, but the age-related increase in effectiveness was nevertheless apparent. Analysis of hospital admissions for fractures, as an NCO in the Cox models indicated the same direction of significant bias in the prior and study periods. However, the effects were closer to the null and not significant after application of the pairwise model suggesting some success in the attenuation of bias.

Information on pneumococcal pneumonia serotypes was unavailable in the absence of routine testing in the UK, which was a limitation of the study. The choice of a composite outcome measure based on antibiotic prescriptions or first hospitalization for suspected pneumococcal pneumonia was less specific than what might be available in smaller studies with access to laboratory-confirmed outcomes and chest X-ray imaging; our outcome was developed with clinician input to reflect the manifestations of pneumococcal disease in clinical practice.

Test-negative case-control studies can greatly reduce misclassification of the outcome by utilising laboratory testing for influenza [37]. This design may also control some confounding bias by restricting the sampling frame to patients requiring laboratory testing following hospital admission [38]. However, this may then reduce the generalizability to more severe outcomes. Furthermore, unmeasured confounding may still persist [39]. Ultimately, the statistical power for the test-negative case-control design to study clinically interesting sub-groups is constrained by the absence of widespread laboratory testing. Two studies using the test-negative case-control design to investigate pneumococcal vaccine effectiveness were able to report serotype-specific vaccine effectiveness [14, 15], but lacked the power to resolve the important question of how PPV23 effectiveness varies by age group. In contrast, our study employed two methods to adjust for unmeasured confounding, and compared these with a high-dimensional adjustment for measured confounders, across subgroups of age. We also employed a sensitivity analysis of an NCO to validate our results. The size of our study enabled a comparison of the effectiveness of PPV23 across age sub-groups.

The finding that effectiveness may increase with age in the two years following vaccination with PPV23 demonstrates an important role mitigating against age-related susceptibility to infection with CAP. While we found a non-significant decrease in effectiveness in the second year after vaccination, the study by Lawrence et al. [15] and the post-hoc analysis of the CAPITA trial [40] found that vaccine effectiveness does not decline with years since vaccination. This may support the vaccination of older adults before the onset of frailty. However, our finding of age-related increase in effectiveness in the two years following vaccination is consistent with those from an investigation into the immunogenic response to PPV23 among Japanese adults≥70y and may support re-vaccination of older adults to booster the immune response to CAP [41].

## Conclusion

The control of pneumococcal pneumonia is a public health priority in countries with an ageing population, such as the UK, because of the higher risk in older age groups. Our study demonstrated a clear reduction in disease burden following the introduction of the UK policy of vaccinating older adults with PPV23. Crucially, we found that the vaccine remained effective, and may even increase in effectiveness, at older ages, supporting the targeting of the oldest old and most frail patients for PPV23 vaccination in order to reduce the burden of pneumococcal disease. The effect of immunosenescence should not be considered in isolation, but has to be set against the increased susceptibility of the oldest age groups when assessing vaccination effectiveness in real world populations. Hence oldest age group may derive the greatest benefit from PPV23, although immunity may nevertheless start to wane soon after vaccination. These findings have implications for the formulation of future pneumococcal vaccination policy in the UK and other countries.

## Supporting information

S1 Table Characteristics of study population for each cohort by pneumococcal vaccination status at cohort entry into study period. (DOCX)

S1 Appendix Supplementary file of codes for exposure and outcomes; adjustment on observed confounders; sensitivity analysis; comparison of first and second year after vaccination. (DOCX)
