# Correction: Real-world effectiveness of pneumococcal vaccination in older adults: Cohort study using the UK Clinical Practice Research Datalink

**DOI:** 10.1371/journal.pone.0314373

**Published:** 2024-11-19

**Authors:** Adam J. Streeter, Lauren R. Rodgers, Jane Masoli, Nan X. Lin, Alessandro Blé, Willie Hamilton, William E. Henley

The first author, Adam J. Streeter, is incorrectly noted as the corresponding author. The correct corresponding author is William E. Henley. William E. Henley’s email address is: w.e.henley@exeter.ac.uk.
