# Supplementary material for: Real-world effectiveness of pneumococcal vaccination in older adults: Cohort study using the UK Clinical Practice Research Datalink
Source: PLoS One. 2022 Oct 13;17(10):e0275642. doi: 10.1371/journal.pone.0275642 (PMC9560513; doi:10.1371/journal.pone.0275642)
Supplement: S1 Table — (DOCX) [file pone.0275642.s001.docx]

| **Cohort** | | **2003** | | **2004** | | | | **2005** | | |
| --- | --- | --- | --- | --- | --- | --- | --- | --- | --- | --- |
| **Treatment group** | | **Vaccinees** | **Controls** | **Vaccinees** | | **Controls** | | **Vaccinees** | **Controls** | |
| Disease registered under Quality Outcomes Framework | AF | 14.3% | 11.9% | 11.0% | 10.6% | | 6.3% | | 6.9% |  |
|  | Asthma | 6.9% | 5.2% | 6.9% | 5.5% | | 7.3% | | 5.6% |  |
|  | Cancer | 9.7% | 8.2% | 9.7% | 8.8% | | 8.4% | | 8.0% |  |
|  | CHD | 21.9% | 18.6% | 19.4% | 16.7% | | 13.3% | | 12.4% |  |
|  | CKD | 6.1% | 3.1% | 19.7% | 12.1% | | 14.8% | | 11.8% |  |
|  | COPD | 5.4% | 5.3% | 5.9% | 5.2% | | 4.4% | | 4.2% |  |
|  | Dementia | 6.7% | 9.0% | 4.7% | 7.1% | | 1.8% | | 3.6% |  |
|  | Depression | 9.8% | 9.0% | 9.4% | 8.7% | | 8.5% | | 7.9% |  |
|  | DM | 6.2% | 4.2% | 8.8% | 6.4% | | 8.4% | | 6.9% |  |
|  | Epilepsy | 1.3% | 1.2% | 1.3% | 1.2% | | 1.2% | | 1.2% |  |
|  | HF | 10.3% | 10.7% | 6.0% | 7.3% | | 2.3% | | 3.9% |  |
|  | Hypertension | 48.5% | 37.5% | 52.1% | 40.9% | | 46.2% | | 37.4% |  |
|  | Hypothyroid | 9.0% | 7.9% | 9.2% | 8.2% | | 7.9% | | 7.2% |  |
|  | Mental Health | 1.0% | 1.6% | 1.2% | 1.6% | | 1.1% | | 1.5% |  |
|  | Stroke | 14.1% | 14.0% | 10.7% | 11.9% | | 5.8% | | 7.6% |  |
| Smoking status | Smoker | 15.4% | 16.4% | 20.9% | 20.6% | | 23.7% | | 25.3% |  |
|  | Not smoker or n/a | 53.4% | 61.4% | 44.9% | 54.1% | | 45.2% | | 49.3% |  |
|  | Ex smoker | 31.2% | 22.2% | 34.2% | 25.3% | | 31.1% | | 25.4% |  |

Table S1: Characteristics of study population for each cohort by pneumococcal vaccination status at cohort entry into study period
