# Supplementary material for: Real-world effectiveness of pneumococcal vaccination in older adults: Cohort study using the UK Clinical Practice Research Datalink
Source: PLoS One. 2022 Oct 13;17(10):e0275642. doi: 10.1371/journal.pone.0275642 (PMC9560513; doi:10.1371/journal.pone.0275642)
Supplement: S1 Appendix — (DOCX) [file pone.0275642.s002.docx]

Real-world effectiveness of pneumococcal vaccination in older adults: cohort study using the UK Clinical Practice Research Datalink - supplementary file

## ICD10 outcome codes

### PPV codes (immstype) in immunisation file:

13, 28

[both codes conditioned on status = 1, i.e: vaccine “given”]

### PPV codes (prodcode) in therapy file:

821, 832, 42612, 42991

### Influenza vaccine codes in immunisation file:

6, 9039, 10821, 12104, 12336, 18330, 18684, 21123, 32942, 35655, 44555, 94301, 95092, 97941, 98047, 98183, 98184, 98217, 98234, 98302, 98303, 98306, 98449

### Influenza vaccine codes in therapy file:

398, 639, 834, 922, 1329, 2139, 2552, 2601, 9710, 10030, 11824, 13595, 16585, 18612, 27407, 30156, 30198, 32391, 38421, 40760, 40876

### ICD10 codes for hospitalisation for suspected pneumococcal pneumonia in HES data:

J13, J15.8, J15.9, J16.8, J17, J18

### Amoxicillin codes in therapy file:

9, 48, 62, 133, 427, 503, 585, 847, 870, 1637, 1722, 1812, 2153, 2281, 3669, 3742, 4154, 7737, 9243, 11613, 11634, 12378, 14371, 14386, 14396, 14407, 15148, 17711, 18786, 21799, 21827, 21829, 21844, 21845, 21963, 22015, 22016, 22017, 22415, 22438, 23238, 23740, 23967, 24150, 24200, 24203, 25484, 26157, 26262, 27714, 27725, 28870, 28872, 28875, 28882, 29337, 29463, 29697, 29858, 30498, 30528, 30743, 30745, 31014, 31286, 31423, 31535, 31661, 31801, 32622, 32640, 32872, 33109, 33110, 33112, 33165, 33222, 33343, 33570, 33689, 33690, 33692, 33696, 33699, 33706, 34001, 34042, 34232, 34384, 34435, 34638, 34679, 34714, 34760, 34775, 34852, 34855, 34857, 34885, 34912, 35570, 36054, 37755, 38684, 40238, 40243, 41090, 41818, 41835

### Doxycycline codes in therapy file:

264, 268, 970, 1046, 2202, 2884, 3152, 6396, 8724, 9267, 10454, 12987, 14904, 15071, 21038, 21828, 21860, 21878, 23405, 23432, 23819, 24126, 24149, 26392, 26747, 30739, 32066, 32419, 33671, 34175, 34300, 34423, 34594, 34765, 40391, 41560, 41605, 46807

### Medcodes from clinical file for symptom descriptions used to qualify antibiotic codes:

| Medcode | Description |
| --- | --- |
| 293 | Respiratory tract infection |
| 4899 | Recurrent chest infection |
| 68 | Chest infection |
| 2581 | Chest infection NOS |
| 3358 | Lower resp tract infection |
| 5534 | Pneumococcal infection |
| 7074 | Respiratory infection NOS |
| 8025 | Acute respiratory infections |
| 14804 | Sputum appears infected |
| 16287 | Chest infection - unspecified bronchopneumonia |
| 17359 | Chest infection - unspecified bronchitis |
| 19400 | Chest infection - pnemonia due to unspecified organism |
| 21061 | Chronic obstruct pulmonary dis with acute lower resp infectn |
| 21113 | Acute respiratory infection NOS |
| 22795 | Chest infection - other bacterial pneumonia |
| 23640 | Other specified acute respiratory infections |
| 3382 | Streptococcal infection |
| 572 | Pneumonia due to unspecified organism |
| 886 | Bronchopneumonia due to unspecified organism |
| 1849 | Lobar (pneumococcal) pneumonia |
| 3683 | Basal pneumonia due to unspecified organism |
| 9639 | Lobar pneumonia due to unspecified organism |
| 10086 | Pneumonia and influenza |
| 11849 | Other specified pneumonia or influenza |
| 12423 | Pneumonia due to streptococcus |
| 13573 | Influenza with bronchopneumonia |
| 22009 | Streptococ pneumon/cause/disease classified/oth chapters |
| 23095 | Bacterial pneumonia NOS |
| 25694 | Pneumonia due to other specified organisms |
| 23333 | Hypostatic pneumonia |
| 24356 | Hypostatic bronchopneumonia |
| 1934 | Laryngotracheobronchitis |
| 1019 | Acute bronchiolitis |
| 17185 | Acute bronchiolitis with bronchospasm |
| 17917 | Acute bronchiolitis NOS |
| 29669 | Acute bronchitis and bronchiolitis |
| 41137 | Acute bronchitis or bronchiolitis NOS |
| 2195 | Bronchiectasis |
| 20364 | Recurrent bronchiectasis |
| 1234 | Productive cough NOS |
| 7708 | Productive cough-yellow sputum |
| 7773 | Productive cough -green sputum |
| 18907 | Cough with fever |
| 8760 | [D]Positive culture findings in sputum |
| 15430 | [D]Sputum abnormal - colour |
| 16026 | Sputum examination: abnormal |
| 24181 | Sputum: mucopurulent |
| 30754 | Yellow sputum |
| 36880 | Green sputum |

## Adjustment on observed confounders

For the purpose of adjusting for confounding, information was collected at baseline on smoking history and comorbidities within the Quality Outcomes Framework (QOF) [1], a scheme to incentivise general practitioners to register common morbidities of patients. The corresponding codes were obtained, with reference to previous work on multimorbidity[2], and supplemented with further codes for conditions identified in an electronic frailty index (eFI)[3] to build a high-dimensional propensity score predicting treatment. Survival times until CAP in each cohort were first regressed on these variables, as well as age and gender. Those variables found to be predictive of the outcome at the 5% level were identified as potential confounders to be included in the propensity score model.

Table S1 shows the degree of imbalance between the vaccine-recipients and controls in the QOF diseases and smoking. The vaccination group was found to have a consistently higher prevalence of diseases registered under the Quality Outcomes Framework compared to the controls. The two leading registered comorbidities were hypertension and coronary heart disease. Hypertension prevalence in the vaccinees ranged from 46% in 2005 to 52% in 2004, and in the controls from 37% in 2005 to 41% in 2004. Similarly, coronary heart disease was more prevalent among the vaccinees with 18.5% compared to 15.6% for the controls from the 2003 cohort falling to 11.3% and 10.5%, respectively, in the 2005 cohort. The proportion of identified smokers was similar between treatment groups, increasing slightly with each cohort.

Stabilised weights were derived from each patient’s propensity score and used to adjust for bias due to measured confounders in an inverse probability treatment weighted (IPTW) Cox regression model of the effectiveness of PPV23 against CAP. We then used the PERR adjustment to mitigate for hidden bias in the results from the IPTW models. This adjusted the IPTW estimates with those from the prior period using stabilized weights predicted from confounders measured at baseline of the prior period. The PERR estimate was calculated as the hazard ratio (HR) of the study period divided by that of the prior period. Bootstrap resampling provided the 95% confidence intervals for the PERR estimate.

From the weighted analysis of the study periods alone adjusting for measured confounders, the greatest effect of vaccination occurred in the 2003 cohort of patients aged 80 and over with an HR of 0.72 (95% CI 0.74 to 0.89) (Table S2). The largest effect, albeit slightly reduced from 2003, was found in the same age group in 2004, with an HR of 0.84 (95% CI 0.77 to 0.92). Effectiveness in the 75-79y old age-group in 2004 and 2005 was found to be similar with HRs of 0.93 (0.95 to 1.01) and 0.90 (95% CI 0.83 to 0.98), respectively. In 2005, the confidence intervals of the estimated HRs 0.90 (95% CI 0.83 to 0.98) and 0.96 (95% CI 0.86 to 1.08) overlapped considerably for the 75-79y old and 80+y age-groups, respectively, while the 65-74y age-group was closest to the null with an HR of 1.03 (95% CI 0.98 to 1.09). However, the weighted estimates for vaccination from the vaccine-free prior periods for all cohorts, except 2003, were greater than one (theoretically these should all be unity in the absence of vaccination). In two age groups of the 2005 cohort, these were significantly greater than one, indicating the presence of significant bias and potentially unresolved confounding that could affect the study period to varying degrees.

| Cohort | Age group | N vaccinated | N controls | HR for each period | | PERR HR |
| --- | --- | --- | --- | --- | --- | --- |
|  |  |  |  | Prior | Study |  |
| 2003 | 80+ | 25870 | 29087 | 0.89 (0.83, 0.96) | 0.72 (0.67, 0.78) | 0.81 (0.74, 0.89) |
| 2004 | 75-79 | 19409 | 16632 | 1.07 (0.98, 1.17) | 0.93 (0.85, 1.01) | 0.86 (0.78, 0.96) |
| 2004 | 80+ | 10619 | 25993 | 1.09 (0.99, 1.19) | 0.84 (0.77, 0.92) | 0.77 (0.68, 0.87) |
| 2005 | 65-74 | 79812 | 49879 | 1.16 (1.10, 1.22) | 1.03 (0.98, 1.09) | 0.89 (0.84, 0.95) |
| 2005 | 75-79 | 15784 | 16403 | 1.07 (0.98, 1.17) | 0.90 (0.83, 0.98) | 0.85 (0.76, 0.94) |
| 2005 | 80+ | 9373 | 25943 | 1.22 (1.11, 1.33) | 0.96 (0.86, 1.08) | 0.79 (0.69, 0.91) |

Table S2: Hazard ratios from the IPTW analysis presented for age-groups of the prior and study periods pertaining to each cohort, and their PERR-adjusted estimates. Age groups, which were incrementally targeted for pneumococcal vaccination from 2003 to 2005, comprised adults aged over 79y; from 75 to 79y; and from 65 to 74y.

Attenuating the weighted estimates of the study periods for each cohort’s age-groups with those of the prior period using the PERR method resolved the 80+ y age-groups to estimates that were in closer agreement, now ranging from 0.77 (95% CI 0.68 to 0.87) in 2004 to 0.81 (95% CI 0.74 to 0.89) in 2003 (Table S2). Similarly, the PERR-adjusted estimates for the 75-79y age-group were also in close agreement, ranging from 0.85 (95% CI 0.76 to 0.94) to 0.86 (95% CI 0.78 to 0.96). Across and within each cohort, an increasing trend in effectiveness with increasing age was evident, the weakest effect with an HR of 0.89 (95% CI 0.84 to 0.95) being evident in the youngest age-group of 65-74y in 2003. Some of the results may have been subject to instability in IPTW analysis, the weights for which were modelled separately for the prior and study periods. However, the evidence of effectiveness and the age related trend in the PERR-adjusted IPTW results was consistent with that from the Pairwise method (Table 2 in main paper).

## Sensitivity analysis

In the sensitivity analysis, the NCO was analysed using the pairwise method in order to assess whether this method could adjust for bias in the analysis PPV23 effectiveness against CAP. The hazard ratios for the effect of the vaccine in the prior and study periods were all significantly below the null, interpreted as a persistent negative bias between the vaccine groups in both periods. Although some fluctuation was evident in the pairwise HRs, none was significantly different from the null, indicating no effect of vaccination on hospital admissions for fractures after successful attenuation of bias (Table S3).

| Cohort | Age group | Hazard ratio (95% CI) of vaccination | | |
| --- | --- | --- | --- | --- |
|  |  | Prior period | Study period | Pairwise adjustment |
| 2003 | 80+y | 0.78 (0.72 ,0.85) | 0.82 (0.77 ,0.88) | 0.90 (0.80 ,1.02) |
| 2004 | 75-79y | 0.67 (0.59 ,0.77) | 0.83 (0.74 ,0.93) | 1.07 (0.88 ,1.31) |
| 2004 | 80+y | 0.85 (0.76 ,0.95) | 0.87 (0.79 ,0.96) | 0.89 (0.76 ,1.05) |
| 2005 | 65-74y | 0.74 (0.67 ,0.82) | 0.82 (0.75 ,0.90) | 1.00 (0.86 ,1.16) |
| 2005 | 75-79y | 0.79 (0.68 ,0.91) | 0.87 (0.77 ,0.99) | 0.96 (0.77 ,1.20) |
| 2005 | 80+y | 0.79 (0.70 ,0.89) | 0.91 (0.82 ,1.00) | 0.97 (0.81 ,1.15) |

Table S3: Pairwise-adjusted hazard ratios for the effect of PPV23 on the negative-control outcome of fractures (excluding thoracic injury).

## Comparison of first and second year after vaccination

The first and second year of each cohort’s study period from the IPTW analysis were analysed separately, each adjusted with the prior period common to each cohort using the PERR method. This produced PPV23 effectiveness estimates specific to each year following vaccination. While the confidence intervals for the first and second year estimates overlapped, those from the second year of each cohort were greater than the first and closer to the null, potentially indicating a waning effect of vaccination (Table S4). The smallest difference was in 2003, with an increase of ~0.06 in the hazard of CAP, equivalent to a reduction of 6% in vaccine effectiveness (VE), while the largest was among the 75-79y age group with a reduction of 14% in VE. While changing hazards over the duration of follow-up could have represented violations of the proportional hazards assumption of the Cox models, these were not readily apparent in the diagnostic plots and so any time-dependent effects would be small relative to each cohort and age-group’s estimate of VE.

| Cohort | Age group | Year of study | Hazard ratio of vaccination |
| --- | --- | --- | --- |
| 2003 | 80+y | 1 | 0.63 (0.56 ,0.70) |
|  |  | 2 | 0.74 (0.66 ,0.84) |
| 2004 | 75-79y | 1 | 0.77 (0.66 ,0.89) |
|  |  | 2 | 0.90 (0.77 ,1.06) |
|  | 80+y | 1 | 0.58 (0.51 ,0.67) |
|  |  | 2 | 0.66 (0.56 ,0.77) |
| 2005 | 65-74y | 1 | 0.83 (0.76 ,0.90) |
|  |  | 2 | 0.91 (0.83 ,1.00) |
|  | 75-79y | 1 | 0.72 (0.61 ,0.84) |
|  |  | 2 | 0.77 (0.65 ,0.92) |
|  | 80+y | 1 | 0.62 (0.54 ,0.72) |
|  |  | 2 | 0.70 (0.59 ,0.82) |

Table S4: Comparison of Pairwise estimates of PPV23 effectiveness against CAP in the first and second year after vaccination

## References

[1] Quality Outcomes Framework n.d. https://digital.nhs.uk/Quality-and-Outcomes-Framework/QOF.

[2] Melzer D, Tavakoly B, Winder RE, Masoli JAH, Henley WE, Ble A, et al. Much more medicine for the oldest old: trends in UK electronic clinical records. Age Ageing 2015;44:46–53. https://doi.org/10.1093/ageing/afu113.

[3] Clegg A, Bates C, Young J, Ryan R, Nichols L, Ann Teale E, et al. Development and validation of an electronic frailty index using routine primary care electronic health record data. Age Ageing 2016;45:353–60. https://doi.org/10.1093/ageing/afw039.
